# Chrononutrition in Gestational Diabetes: Toward Precision Timing in Maternal Care

**DOI:** 10.3390/jpm15110534

**Published:** 2025-11-03

**Authors:** Viktoria Xega, Jun-Li Liu

**Affiliations:** 1 MeDiC Program, The Research Institute of McGill University Health Centre, Montreal, QC H4A 3J1, Canada; jun-li.liu@mcgill.ca; 2 Division of Endocrinology and Metabolism, Department of Medicine, McGill University, Montreal, QC H4A 3J1, Canada

**Keywords:** chrononutrition, gestational diabetes, circadian rhythm, precision nutrition, meal timing, glycemic control, maternal care, digital health, chronotype, time-restricted eating

## Abstract

Gestational diabetes mellitus (GDM) is a heterogeneous disorder that compromises maternal and offspring health. Conventional medical nutrition therapy focuses on nutrient composition and caloric targets but largely omits timing and individualized biology. This narrative review synthesizes mechanistic, epidemiologic and interventional evidence linking circadian biology and meal timing (chrononutrition) to maternal glycemic control. Observational cohorts associate late eating and breakfast skipping with worse glycemia, while pilot interventions and CGM-based studies indicate that front-loading carbohydrates, restricting evening carbohydrate, extending overnight fasting (≈10–12 h), and simple within-meal sequencing can reduce postprandial excursions and increase time-in-range. We propose a pragmatic, tiered clinical pathway in which routine second-trimester triage (50 g glucose challenge test and ultrasound abdominal subcutaneous fat thickness) identifies higher-risk women for short-term CGM phenotyping and prioritized chrononutrition counseling. Integrating phenotype-matched timing interventions with dietetic support and digital decision tools allows rapid, individualized adjustments informed by real-time glucose patterns and patient chronotype. In principle, this tiered strategy could improve daily glycemic profiles, reduce the need for pharmacotherapy, and translate into better neonatal outcomes if supported by larger randomized trials. Chrononutrition therefore offers a promising extension of standard care: simple, low-cost adjustments to “when” food is eaten, supported by digital tools, could allow nutrition therapy for GDM to become more precise, more responsive, and ultimately more effective for both mother and child. Key priorities include validating bedside and chrono-omic stratifiers, testing scalable delivery platforms, and ensuring equitable access to personalized chrononutrition in pregnancy.

## 1. Introduction

Gestational diabetes mellitus (GDM), defined as increased glucose levels first diagnosed during pregnancy [1], is a common pregnancy complication, affecting approximately 3–15% of pregnancies globally [2,3]. Its clinical relevance extends beyond the gestational period, as women diagnosed with GDM face elevated risks for developing type 2 diabetes and cardiovascular disease, while their offspring are more likely to experience macrosomia, early obesity, and glucose intolerance [4,5,6].

Despite its common definition, GDM is a heterogeneous condition. MTNR1B (melatonin receptor 1B) encodes a melatonin receptor that modulates circadian signaling and insulin secretion; common *MTNR1B* variants (like rs10830963) associate with higher fasting and post-challenge glycemia and increased GDM risk [7,8]. TCF7L2 (transcription factor 7-like 2) encodes a regulator of incretin signaling and β-cell function; *TCF7L2* variants (for example rs7903146) are among the strongest genetic risk loci linking dysglycemia and GDM [9,10]. Along with these genetic variants, circadian phenotypes (chronotype), and gut microbiome shape disease course and response to intervention [11,12,13]. This partly explains why some control GDM with lifestyle changes alone, while others need more treatment. Yet, most current diet therapy is population-based. Medical nutrition therapy (MNT) typically emphasizes macronutrient balance, glycemic index, and caloric goals. It aims to optimize maternal glucose control, promote healthy gestational weight gain, and support fetal growth and development [14]. MNT alone offers little guidance on when food should be consumed or how individual biology might shape response.

As a result, precision nutrition remains largely aspirational in GDM care. A growing body of evidence suggests that circadian timing, particularly the alignment between food intake and internal biological clocks, plays a crucial role in metabolic regulation. This has given rise to the concept of chrononutrition: the study of how the timing of meals influences metabolic outcomes through circadian pathways [15]. Early findings suggest that front-loading caloric intake earlier in the day, maintaining consistent overnight fasting windows, and tailoring eating patterns to an individual’s chronotype may improve glycemic control during pregnancy.

In this context, personalized nutrition has emerged as a promising frontier in maternal healthcare. One particularly exciting and underexplored area is chrononutrition (the study of how the timing of food intake interacts with circadian rhythms). Early evidence suggests that both meal timing [16,17,18,19] and molecular phenotypes [20,21,22] may play crucial roles in glucose regulation during pregnancy. These findings offer new opportunities for precision intervention.

Advances in digital health tools create new opportunities. Devices such as continuous glucose monitoring (CGM), wearables, sleep trackers and app-based food logs can help apply these strategies in real-world settings. Machine learning algorithms and, in the future, molecular phenotyping can further support adaptive, data-driven nutrition care.

This review focuses on the application of circadian principles to the nutritional management of GDM. First, we summarize the physiological basis for chrononutrition in pregnancy. Next, we review human studies linking meal timing to glycemic outcomes. Finally, we outline emerging strategies for implementing personalized, time-sensitive dietary care in clinical practice.

## 2. Methods

We performed a narrative review with targeted evidence synthesis to summarize clinical and mechanistic literature on chrononutrition in gestational diabetes. We searched PubMed/MEDLINE, Embase, Web of Science, and ClinicalTrials.gov up to July 2025. We used combinations of terms, including “gestational diabetes,” “chrononutrition,” “meal timing,” “time-restricted eating,” “chronotype,” and “continuous glucose monitoring.” Titles and abstracts were screened for relevance. Full texts were reviewed where necessary. We included human clinical studies (observational and interventional), key mechanistic studies, and recent reviews addressing meal timing and circadian biology in pregnancy. For each included paper, we extracted study design, population, exposures or interventions, sample size, and primary outcomes. We synthesized findings thematically. A total of 12 human studies directly testing chrononutrition interventions in GDM (9 observational, 3 interventional) were identified and are summarized in Table 1. To cover translational aspects, we also included terms on guideline development, regulatory approval of digital tools, and patient engagement in dietary and digital interventions. Because this is not a systematic review, we did not perform formal risk-of-bias scoring or meta-analysis. Instead, we synthesized the findings narratively, grouping them into four domains: mechanistic, observational, interventional, and clinical translation.

## 3. Clinical Nutrition in GDM: What We Know and Do No Not Know

Gestational diabetes mellitus (GDM) presents a growing clinical and public health challenge, with significant implications for both maternal and fetal outcomes [3,14,23,24,25]. Poorly controlled hyperglycemia during gestation is associated with an elevated risk of hypertensive disorders, fetal overgrowth, increased rates of cesarean delivery, and the programming of long-term cardiometabolic diseases in the offspring [26,27].

Medical nutrition therapy (MNT) remains the cornerstone of GDM management [3,28]. It is widely endorsed as a first-line intervention by major professional organizations, such as American Diabetes Association (ADA) and the International Federation of Gynecology and Obstetrics (FIGO) [3,14]. Typically, this includes personalized caloric recommendations and a macronutrient distribution of approximately 40% carbohydrate, 35% fat, and 25% protein, with adjustments made according to postprandial glucose responses [29,30]. Emphasis is placed on low-glycemic-index foods, which have demonstrated efficacy in reducing postprandial glucose spikes and potentially lowering the need for insulin [31]. Meal planning often involves dividing caloric intake into three moderate meals and two to three snacks per day to promote glycemic stability. More recently, dietary patterns such as the Mediterranean and DASH (Dietary Approaches to Stop Hypertension) diets have been incorporated into GDM care, given their anti-inflammatory effects and beneficial impacts on glucose regulation [32,33,34,35].

Yet despite these guidelines, many individuals with GDM do not achieve glycemic targets through diet alone. Clinical heterogeneity plays a significant role. Some women require pharmacological treatment even with good adherence. Contributing factors include differences in insulin resistance severity, beta-cell function, genetic background, and lifestyle. Ethnic and cultural differences, dietary customs, and access to nutritious foods further compound variability. For example, genetic and metabolic studies suggest that insulin secretion and carbohydrate tolerance may vary by ethnicity and genomic background [36,37]. These findings emphasize the importance of moving beyond a standardized dietary approach toward more individualized models of nutritional care.

Moreover, MNT is still largely standardized, designed for the “average” patient. This leaves little room for tailoring based on individual biology or behavioral context. Cultural practices, such as meal timing and composition, often conflict with generalized dietary advice [38]. In many cases, health literacy or numeracy limitations hinder the ability of patients to understand and apply nutritional principles such as glycemic index or carbohydrate counting [39]. Pregnancy-related symptoms such as nausea, altered taste preferences, and fatigue can also interfere with consistent adherence to structured dietary regimens. Moreover, limited access to trained dietitians or comprehensive nutritional counseling, particularly in under-resourced settings, further reduces implementation fidelity.

A critical limitation of current nutritional guidelines is their lack of temporal and molecular personalization. While considerable attention is paid to what and how much to eat, very little guidance is available on when to eat—even though accumulating evidence links circadian alignment with improved glycemic control. Similarly, nutritional recommendations remain phenotype-agnostic, with no stratification based on insulin resistance profiles, chronotype, or molecular biomarkers such as metabolomic or epigenetic signatures. The omission of meal timing and individual molecular traits may limit the effectiveness of current nutritional interventions in GDM. Even simple strategies like modifying the sequence of macronutrient intake have demonstrated clinically meaningful effects but remain absent from clinical protocols. In a clinical trial by Murugesan et al. (2025) [40], women with GDM managed by diet or oral hypoglycemic agents followed a structured food order strategy (fiber first, then protein, followed by carbohydrates) for ten days, with glucose continuously monitored using CGM. Compared to a baseline period on their usual diet, this sequencing approach led to significant reductions in postprandial glucose excursions [40]. These results highlight that meal structuring, not just composition, can enhance MNT efficacy and represent an overlooked opportunity in existing dietary protocols for GDM. To illustrate the limitations of a generalized approach, consider the following clinical scenarios in Box 1

Box 1 **Why MNT is not one size fits all: timing focused case scenarios.**

- **Patient A** is a South Asian woman with early morning hyperglycemia despite a low-carbohydrate evening meal; further exploration reveals late-night snacking and a strong evening chronotype.
- **Patient B**, who is obese, presents with minimal postprandial glucose excursions but persistently elevated fasting glucose levels—suggesting a need for targeted nocturnal nutritional adjustments.
- **Patient C**, a Latina woman, follows culturally normative patterns of late dinners and small breakfasts. Her glycemic control improves only after redistributing caloric intake earlier in the day.

These examples reflect the multifaceted interplay between biology, behavior, and cultural context that current guidelines often fail to address.

## 4. Chrononutrition in Pregnancy and GDM

### 4.1. Circadian Physiology in Pregnancy

The circadian system is orchestrated by the suprachiasmatic nucleus (SCN) in the hypothalamus, which synchronizes behavioral and physiological rhythms, including feeding, sleep, and hormone release to the 24 h light–dark cycle [41,42,43]. This central pacemaker is complemented by peripheral clocks located in nearly all tissues, including liver, adipose, pancreas, and placenta. These clocks are entrained not by light, but by behavioral cues like feeding and activity, regulating rhythmic gene expression involved in glucose uptake, insulin secretion, lipid metabolism, and energy balance [44,45]. During pregnancy, circadian coordination becomes more complex. Hormonal shifts, particularly in estrogen, progesterone, and placental hormones, modify sleep architecture, appetite regulation, and insulin sensitivity [46]. Evidence from animal models suggests that pregnancy induces phase shifts in peripheral clock gene expression, possibly to support altered metabolic demands [47,48]. In humans, data are more limited, but there is growing evidence that circadian disruption may impair placental function, alter fetal programming, and reduce maternal insulin sensitivity [46]. BMAL1 (ARNTL; brain and muscle ARNT-like 1) and PER3 (period circadian regulator 3) are core molecular clock components: BMAL1 forms part of the activating transcriptional complex that drives rhythmic gene expression in metabolic tissues, while PER3 participates in the negative-feedback arm that shapes circadian phase and amplitude [49,50]. In peripheral blood of pregnant women with GDM, BMAL1 and PER3 transcript levels are significantly reduced in amplitude compared to controls, correlating with elevated HbA1c and HOMA-IR [51]. These human data underscore the clinical relevance of circadian disruption and the need to align meal timing with maternal molecular rhythms.

Chrononutrition refers to the timing of food intake about the body’s circadian clock, recognizing that when one eats can be as important as what or how much is eaten [52]. Key terms in the field of chrononutrition are defined in Box 2 to guide interpretation.

Under normal conditions, metabolic processes like glucose tolerance and insulin secretion follow a daily rhythm: humans are generally most insulin-sensitive in the morning and become more insulin-resistant by evening [53,54]. Eating at biologically inappropriate times (such as during the usual sleep phase at night) can desynchronize internal clocks—the central clock in the brain versus peripheral clocks in metabolic organs—leading to circadian misalignment [52,55]. Such misalignment adversely affects metabolism and has been linked to increased risks of obesity and diabetes [52].

Box 2 **Chrononutrition concepts and terminology.**

- **Circadian rhythm:** The ~24-hour internal biological clock regulating physiology and behavior (including hormone release, metabolism, and the sleep–wake cycle) in response to light–dark changes [56,57].
- **Chronotype:** An individual’s characteristic timing of sleep and activity (often “morning” vs. “evening” types). It reflects personal circadian phase: “early” chronotypes wake/sleep earlier (shorter intrinsic cycle) and “late” chronotypes prefer later hours [15].
- **Chrononutrition:** The study of how meal timing and dietary composition interact with the circadian system. This includes timing of food intake and specific nutrients that can synchronize or disrupt molecular clocks [15].
- **Time restricted eating (TRE):** A dietary approach in which all daily caloric intake is restricted to a consistent window of 4 to 12 hours each day, without specific guidelines on calorie or nutrient restriction during that period. The remaining hours are spent fasting. TRE aims to align eating patterns with circadian rhythms and typically does not require changes to the amount or quality of food consumed, only the timing of intake [58,59].

During pregnancy, maternal physiology undergoes circadian adaptations. There are shifts in clock gene expression across gestation that modulate the expression of metabolic genes (e.g., those governing gluconeogenesis and insulin signaling) to meet the energy demands of the growing fetus [47,52,60]. If these finely tuned rhythms are disrupted, a pregnant woman may be predisposed to metabolic dysregulation, reduced insulin sensitivity, and impaired glucose homeostasis. In essence, pregnancy might be a state in which circadian coordination is especially critical for maintaining optimal insulin action and energy balance.

Emerging evidence supports the biological rationale that circadian disruption can impact pregnancy outcomes. Pregnant women engaged in shift work (especially night shifts) experience higher rates of miscarriage, preterm birth, low birth weight, and hypertensive disorders [52,61,62,63,64]. Specifically, with respect to GDM, circadian disruption appears to be a notable risk factor. A large prospective study of over 10,000 first-time pregnancies reported that evening and night shift work during pregnancy was associated with approximately 75% higher odds of developing GDM compared to daytime work [65]. Notably, in that analysis, the link between shift work and GDM was partly mediated by irregular sleep timing, accounting for approximately a quarter of the excess risk [65]. These data underscore how modern lifestyle factors, from late-night eating to variable sleep schedules, can misalign circadian rhythms and potentially impair glucose regulation in pregnancy.

### 4.2. Human Studies on Meal Timing, Chronotype, and GDM

Observational studies over the past decade have increasingly pointed to the importance of not just what *t* women eat during pregnancy, but when *n* they eat it. Chronotype, an individual’s preference for morning or evening activity, may play a role in metabolic health during pregnancy. In a cohort of 305 women with GDM in Brazil, only 7% were identified as having an “evening” chronotype, yet this small subgroup had disproportionately worse outcomes [11]. GDM patients with an evening preference had significantly poorer sleep quality and higher rates of insomnia and fatigue, and importantly, they were far more likely to develop preeclampsia and to have their newborn require NICU admission than women with a morning chronotype. In fact, evening-type GDM mothers had an adjusted odds ratio ~4 for neonatal intensive care unit (NICU) need and ~3 for preeclampsia compared to morning-type mothers. The investigators noted that eveningness was an independent risk factor even after controlling for baseline hypertension and sleep quality, highlighting a potential intrinsic circadian vulnerability [11]. One interpretation is that women with an evening chronotype tend to keep later hours (later meals and later sleep), misaligning their food intake and hormone cycles with the endogenous circadian insulin sensitivity peak. This misalignment could exacerbate the metabolic stress of pregnancy and GDM. It also mirrors findings in non-pregnant populations where “night owls” have higher rates of metabolic syndrome and diabetes [65,66,67], presumably due to behaviors like late eating and reduced sleep that accompany an evening orientation.

Meal timing patterns themselves have been linked to GDM risk. Perhaps the clearest example is breakfast consumption, which anchors the daily feeding–fast cycle. A large Japanese prospective study found that women who frequently skipped breakfast had higher odds of developing gestational diabetes. In over 84,000 pregnancies, those who ate breakfast only 0–2 days per week (vs. daily eaters) showed a 21% increased odds of GDM (adjusted OR 1.21, 95% CI 1.05–1.41) [68]. Even moderate infrequency (eating breakfast 3–4 days per week) trended toward an elevated risk. The authors concluded that consuming breakfast less than three days a week before and during early pregnancy was associated with a significantly higher incidence [68]. Skipping breakfast may lead to greater hunger and larger meals later in the day, which, coupled with the circadian drop in insulin sensitivity that occurs in the evening, creates a metabolic disadvantage. It may also shorten the overall overnight fasting interval and decrease the normal morning rise in insulin action, both of which can worsen glycemic control. This epidemiologic insight reinforces the clinical adage that “breaking the fast” each morning is metabolically beneficial, especially for expecting mothers, by aligning nutrient intake with the body’s daytime metabolic preparedness.

Conversely, late-night eating and irregular meal schedules have been associated with poorer glycemic measures in pregnancy. A cross-sectional study within a Singaporean pregnancy cohort of 1061 pregnant women examined 24 h dietary patterns of glucose levels at 26–28 weeks’ gestation [69]. Women who prolonged their eating into late-night hours (thereby having a shorter overnight fasting window) tended to have higher glucose levels. In contrast, those who fasted for longer durations overnight had better glycemic profiles. Specifically, each additional hour of nightly fasting (between 7 PM and 7 AM) was associated with a small but significant reduction in fasting glucose by ~0.03 mmol/L [69].

Meanwhile, a greater number of eating episodes per day (grazing or frequent snacks) was linked to higher 2 h postprandial glucose levels (each extra eating episode corresponded to a 0.15 mmol/L increase in 2 h glucose) [69]. In adjusted models, longer uninterrupted nighttime fasts predicted lower fasting sugar, and fewer daily meals predicted lower post-meal sugar, suggesting that a concentrated, aligned eating pattern (three meals in a defined window) might be metabolically advantageous in pregnancy. These findings are in line with chrononutrition principles: continuous grazing or midnight snacking imposes a metabolic strain, whereas time-aligned eating (with sufficient fasting overnight) allows the circadian metabolic machinery to reset and function optimally for the next day [69]. For pregnant women, adopting regular meal times like including a hearty breakfast and avoiding late dinners, could improve glycemic responses. This is a practical discovery.

Importantly, studies focusing on women already diagnosed with GDM show that chrononutrition factors can distinguish better versus worse glycemic control. In a prospective analysis of 208 Israeli women with gestational diabetes, meal timing and composition were strongly associated with the mother’s glucose levels and even infant outcomes [70]. Women who habitually ate a late breakfast (significantly delaying their first daily meal) had more difficulty achieving glucose targets, with over twice the risk of suboptimal glycemic control compared to those who ate earlier in the day [70]. Moreover, a higher proportion of each day’s calories (particularly carbohydrates) consumed in the evening hours was correlated with worse maternal glycemic control (RR for poor control ~1.2 per incremental increase in evening carb intake) [70]. These results make sense given that eating a carb-rich meal at night (when insulin sensitivity is lowest) will tend to produce larger glucose excursions. Intriguingly, Messika and colleagues also noted effects on fetal growth: in GDM mothers, greater carbohydrate intake in both the morning and evening was associated with giving birth to a larger infant (neonatal birth weight >85th percentile) [70]. One might expect evening intake to matter (as excess nighttime calories could promote fetal overnutrition when maternal glucose is less controlled), but the morning carb effect suggests a more complex relationship, possibly that a very high carb load at any one time of day can overwhelm glycemic regulation in GDM.

Together, these findings emphasize that both intrinsic circadian biology (chronotype) and modifiable behaviors (meal timing, fasting intervals) contribute meaningfully to maternal glucose dynamics. As with the general population, pregnancy appears to amplify the consequences of circadian misalignment.

### 4.3. Translational Implications and Interventions

A growing number of trials are translating these insights into testable interventions. One promising strategy is to intentionally align meal timing with the body’s circadian glucose metabolism to improve glycemic control in GDM. For example, a recent randomized controlled trial tested the impact of a chrononutrition-focused intervention in women with gestational diabetes [13]. In this trial, 103 women with GDM were randomized either to standard care alone or to an intervention combining tailored meal timing guidance plus sleep hygiene counseling on top of standard GDM care. The chrononutrition arm encouraged participants to consume more of their calories earlier in the day, avoid heavy late-evening meals, and maintain consistent sleep–wake times. The results were encouraging: the intervention significantly improved maternal glycemic control, reducing the proportion of women with suboptimal glucose readings (defined as <80% of self-monitored values within target range) by a substantial margin. After adjusting for confounders, the risk of poor glycemic control was ~72% lower in the chrononutrition + sleep group compared to controls. Notably, investigators observed that lowering carbohydrate intake in the evening was the key factor mediating this improvement—the intervention group curbed nighttime carbs and subsequently had much better glucose profiles [13]. This RCT did not find a significant difference in newborn outcomes such as incidence of large-for-gestational-age infants but it was relatively small and perhaps not powered for neonatal endpoints. What it did demonstrate is the feasibility and efficacy of integrating circadian-friendly eating patterns into GDM care to enhance maternal glycemic control. In essence, it provides proof-of-concept that lifestyle interventions targeting when mothers eat (and sleep) can complement the traditional focus on diet quality and quantity.

A second trial by Murugesan et al. (2025) focused on intra-meal sequencing. Women with GDM (n = 27) were guided to consume fiber-rich foods first, then protein, then carbohydrates for 10 days following a baseline period [40]. This behavioral intervention, combined with mobile health support, resulted in meaningful glycemic improvements [35]. The simplicity and scalability of this approach, requiring no change in total food content or caloric intake, suggest that food sequencing could be a low-burden, high-impact tool for refining dietary management in GDM, particularly in digitally supported care models.

Other translational research is exploring circadian interventions in pregnancy. For instance, addressing sleep disturbances, which frequently coexist with circadian misalignment, may improve metabolism. Small trials of sleep extension or improved sleep timing in women with GDM have hinted at better fasting glucose and lower insulin resistance [71]. These studies recognize that sleep and meal timing are interlinked pillars of circadian health; a synchronized approach (encouraging earlier dinners and adequate overnight sleep) could synergistically improve outcomes. A consolidated summary of these human chrononutrition studies in GDM is provided in Table 1.

**Table 1:** Human studies on chrononutrition and GDM (2015–2025).

Study (Design and Population)	Chrononutrition Exposure/Intervention	Main Findings
Chandler-Laney et al. (2016)—Observational (n = 40, stratified by BMI) [72].	Late-night carbohydrate intake (3rd-trimester food diaries).	In women with obesity: higher nighttime carbohydrate intake → higher 2 h OGTT glucose and lower insulin secretion.
Loy et al. (2017)—Cross-sectional (n = 1237–1061 completed) [70].	Meal frequency and overnight fasting duration.	Shorter overnight fasting and more frequent eating → higher maternal glucose concentrations
Deniz et al. (2019)—Cross-sectional (n = 148) [73]	Night eating syndrome (NES) vs. no NES.	NES → higher fasting insulin, HOMA-IR, and HbA1c.
Dong et al. (2020)—Prospective cohort (n = 84,669 pregnancies—1935 cases of GDM) [69].	Breakfast frequency (skipping vs. eating)	Skipping breakfast before or during early pregnancy → higher GDM risk (OR ≈ 1.21).
Rasmussen et al. (2020)—Randomized crossover (n = 12) [74].	High vs. low morning carbohydrate intake.	Higher morning carbohydrate intake → lower average glucose, despite modest rise in variability
Morris et al. (2019)—Prospective observational pilot (n = 200; 101 completed) [75].	Meal and snack frequency/distribution.	Three meals + three snacks/day → better glycemic control, especially fasting glucose, vs. lower-frequency eating.
AlMogbel et al. (2022)—Retrospective cohort (n = 345) [76].	Ramadan fasting duration and timing.	Increased neonatal hyperbilirubinemia, decreased neonatal hypoglycemia, birth weight unaffected
Facanha et al. (2022)—Cross-sectional (n = 305) [17].	Chronotype classification.	Evening chronotype → higher risk of preeclampsia and NICU admission
Yong et al. (2022)—Intervention trial (n = 12—but 10 completed the study) [77].	Meal sequencing and meal frequency (five patterns tested: carbs first, protein/veg first, soup first, 3 meals vs. 6 meals)	Protein/vegetables first or soup first → lower mean and peak glucose vs. carbs first; Carb-first meals → larger excursions; increasing meal frequency (6 vs. 3 meals/day) → reduced peaks and excursions at equal calories.
Murugesan et al. (2025)—Interventional pilot (n = 27) [40].	Meal sequencing (vegetables/protein first, carbohydrates last) with short-term CGM feedback to individualize advice.	Reduced postprandial excursions, ↑ CGM time-in-range, ↓ glycemic variability vs. standard dietetic advice
Nakano et al. (2025)—Cross-sectional (n = 144) [78].	Overnight fasting duration and meal frequency.	Longer overnight fasting → lower glycated albumin
Messika et al. (2024)—Prospective cohort (n = 208, GDM) [79].	Breakfast timing, evening carbohydrate intake, sleep quality.	Late breakfast + high evening carbohydrate intake → poor glycemic control, ↑ LGA risk

Abbreviations: GDM—gestational diabetes mellitus; OGTT—oral glucose tolerance test; LGA—large for gestational age; NES—night eating syndrome; HOMA-IR—homeostatic model assessment of insulin resistance; NICU—neonatal intensive care unit; ↑ increase; ↓ decrease.

## 5. Translating Chrononutrition into Practice

Building on the mechanistic and epidemiological evidence reviewed above, clinicians can integrate simple, time-based strategies into GDM management. Below we outline key approaches and illustrate them with real-world examples.

### 5.1. Front-Loading Carbohydrates

Because insulin sensitivity peaks in the morning and declines by evening, concentrating carbohydrate intake earlier in the day can improve glycemic control. In the Israeli CGM-based trial, women assigned to a chrononutrition intervention consumed most of their carbohydrates before 3 PM and reduced evening carbs; this shift accounted for the 72% lower risk of suboptimal glucose readings compared with standard care [71,72].

### 5.2. Consistent Overnight Fasting

Extending the nightly fasting interval allows endogenous metabolic pathways to reset. Data from a Singapore cohort showed that each additional hour of overnight fasting (7 PM–7 AM) correlated with a 0.03 mmol/L reduction in fasting glucose, while shorter fasts and frequent snacking were linked to higher postprandial levels [70]. A pragmatic target of a 10–12 h fast from last meal to first balances feasibility with metabolic benefit.

### 5.3. Meal Sequencing

Altering within-meal order provides another low-burden intervention. In a 15-day CGM pilot, participants ate vegetables first, then protein, and carbohydrates last; this simple reordering reduced postprandial excursions by 20–30% without changing total caloric intake [77]. Real-world cases illustrate how chrononutrition can be applied to improve glycemic outcomes in GDM (see Box 3).

Box 3 **Case vignettes: applying chrononutrition in GDM.**

- **Patient A (28 y, 26 wk gestation)**

Despite a low-carbohydrate dinner, her CGM revealed marked post-dinner spikes and elevated fasting glucose. Late-night snacking and an evening chronotype were identified. By shifting breakfast to 7 AM and completing all meals by 7 PM, she achieved a 20% reduction in time above range within one week.

- **Patient B (32 y, 24 wk gestation)**

Routine family dinners at 9 PM shortened her overnight fast to <8 h, leading to high pre-meal glucose levels. Moving her main meal to 6 PM and adding a light, fiber-rich breakfast improved both fasting and post-breakfast glycemia.

- **Patient C (35 y, 28 wk gestation)**

As a rotating shift worker, she had no consistent meal times, resulting in erratic glucose excursions. Establishing a 10 h eating window aligned to her active periods, regardless of clock time, stabilized her CGM profile and improved sleep quality.

Practical, clinician-facing chrononutrition strategies and the level of supporting evidence are summarized in Table 2.

For detailed clinician-oriented food group and timing guidance (including stage applicability for GDM), see Table A1 (appended before the References).

## 6. Integrating Molecular and Digital Tools for Precision Maternal Care

Emerging biomarkers and real-time monitoring enable the customization of chrononutrition strategies to address individual patient requirements, moving beyond uniform timing recommendations.

### 6.1. Molecular Stratification and Triage

Molecular stratification has revealed heterogeneity in GDM pathophysiology, allowing more precise identification of chrono-sensitive phenotypes. Multi-omics technologies, including transcriptomics, metabolomics, and microbiomics, have unraveled time-dependent biological signatures: Genomic data (maternal and fetal) can identify variants affecting glucose metabolism or circadian regulation, informing risk stratification [85,86]. Epigenetic marks may reveal in utero exposure, while transcriptomics/proteomics (e.g., in placenta) can show active pathways [87,88]. Metabolomics (blood/urine) captures real-time metabolic state, including glucose, lipids and hormone rhythms [89]. The gut microbiome is also important: diet-induced changes in microbial taxa and metabolites (e.g., short-chain fatty acids) influence insulin sensitivity and may mediate chrononutrition effects [90]. These insights suggest that traditional GDM classifications, based solely on glucose levels, fail to account for underlying chrono-biological diversity. Stratifying patients using temporal omics markers could allow clinicians to tailor interventions with greater specificity.

In addition to molecular approaches, simple clinic-based predictors may aid early triage. For example, combining the 50 g glucose challenge test (GCT) with ultrasound-measured abdominal subcutaneous fat thickness (ASFT) at 24–28 weeks has been shown to predict GDM risk with good sensitivity and specificity [91]. Integrating such anthropometric–biochemical markers with meal timing strategies enables a pragmatic, tiered pathway: women flagged as high-risk could be prioritized for early chrononutrition counseling and short-term CGM phenotyping, with intensified, tailored meal timing interventions if CGM shows nocturnal hyperglycemia or large postprandial excursions [91].

### 6.2. Wearables and Real-Time Monitoring

Continuous glucose monitors (CGMs) and wearable trackers form the bridge between circadian behavior and molecular profiling. CGMs provide minute-by-minute glucose readings across the day, revealing patterns (fasting, postprandial peaks) that standardized tests often miss [92]. In pregnancy, CGM studies have shown that even before formal GDM diagnosis, women who will develop GDM have higher mean glucose and more time above normal range [92]. This continuous data allows fine-tuning of meal timing: for instance, if late-night snacks consistently spike glucose, algorithms can advise earlier or smaller dinners. Similarly, wearable devices (actigraphy watches, smartphone apps) record sleep onset/offset and light exposure, quantifying chronotype and circadian alignment. Poor sleep quality and late meals have been linked to worse glycemia and larger birth weight in GDM [79]. Mobile apps can log food intake, macronutrients and meal times, which combined with CGM create a precise diary of “chrononutrition.” Although most current GDM apps simply record inputs or give generic advice [93], the vision is an intelligent platform: an AI-enabled app that collects data from CGM and sensors, analyzes an individual’s circadian-metabolic profile, and then generates dynamic recommendations (e.g., “shift carbs to breakfast,” “increase overnight fasting”). Notably, recent work developed an ML model predicting postprandial glucose response in GDM using CGM, diet logs, and microbiome data, finding that including microbiota features significantly improved prediction accuracy over carbohydrate counting alone [90]. Such tools demonstrate that integrating wearables and omics through AI can power precision nutrition.

### 6.3. AI-Driven Decision Support

An integrated chrono-omics pipeline requires advanced analytics. Machine learning algorithms can sift through genomic variants, metabolite levels, microbiome profiles and daily routines to identify predictors of glycemic excursions or dietary success. Some studies have already applied ML to GDM risk prediction, emphasizing the need to tailor models to specific populations [94]. In practice, incoming patient data (genome, lab tests, CGM traces, meal timings) would feed a trained AI model that outputs a personalized meal plan: for example, earlier breakfast time and reduced evening carbohydrates if the patient’s pattern shows nocturnal hyperglycemia and late chronotype. The plan might also include probiotic or prebiotic foods to modify a dysbiotic microbiome, or micronutrient suggestions based on genomics (e.g., vitamin D dosing if polymorphisms affect its metabolism). Most importantly, the system operates in a feedback loop: real-time monitoring (CGM, sleep tracker) validates the impact of the recommendations, allowing iterative adjustments.

Implementation and limitations. While the potential is high, practical deployment faces hurdles. Multi-omics assays and AI analytics are still largely research tools, not routine clinical tests. Ensuring data quality (accurate food logging, adequate biospecimen collection) is challenging. Existing mHealth apps for GDM are mostly basic—very few incorporate AI-driven decision support [94]—highlighting a technology gap. Moreover, AI models trained on one demographic may not generalize; indeed, studies show GDM risk predictors vary by ethnicity and underscore the need for diverse training datasets [94]. Feasibility is constrained by cost, data privacy concerns, and the need for user-friendly interfaces. Any recommendations must be validated: randomized trials are needed to prove that a chrono-omics strategy improves outcomes beyond standard care. As Liu et al. (2025) note, large, ethnically diverse cohorts and rigorous methodology are essential before integrating omics into routine pregnancy care [95]. In summary, an integrated chrono-omics framework, linking SCN-driven rhythms with genomics, metabolomics and real-time monitoring, promises to personalize nutritional therapy for GDM. Realizing this vision will require multi-disciplinary collaboration. To illustrate how chrono-omics can be applied in clinical care, we present a real-world vignette integrating molecular profiling, digital monitoring, and behavioral coaching (see Box 4).

Box 4 **Tailoring meal timing and lifestyle strategies in GDM management.**

A 30-year-old woman (“A.S.”) at 26 weeks’ gestation is diagnosed with GDM on routine screening. She has a BMI of 32 and a family history of type 2 diabetes. In our precision nutrition program, A.S. provides a one-time blood sample for genomic analysis and a stool sample for microbiome sequencing. Genetic testing reveals variants associated with insulin resistance, and her gut microbiome shows low levels of Bifidobacterium. She is fitted with a CGM and a wearable sleep/activity tracker, and logs meals via a smartphone app. Over the next week, data show that A.S. habitually skips breakfast until 9 AM and consumes most of her carbs after 8 PM, and reports poor sleep quality. The CGM confirms postprandial spikes and elevated overnight glucose. An AI algorithm integrates these findings with her omics profile: recognizing that late eating and poor sleep are linked to suboptimal glycemia [95], it generates a personalized plan. A.S. is coached to move breakfast to 7 AM (even if light) and dinner by 7 PM, shifting ~20 g of carbohydrates from evening to morning (a strategy shown to lower fasting glucose in GDM) [95]. Her dietitian prescribes a high-fiber morning meal (to nourish beneficial gut microbes) and a modest dinner, and recommends sleep hygiene measures. Over the next week, CGM data show a tighter glucose profile (time-in-range improves) and sleep-tracker data confirm longer uninterrupted sleep. A.S. reports feeling more energetic in the morning. Her obstetrician notes that, unlike her prior family history, her fasting glucose normalizes and insulin is not needed. This vignette illustrates how linking meal timing, wearable data and molecular insights can produce tailored dietary advice.

### 6.4. Translational Implications: From Bench to Bedside

#### 6.4.1. Clinical Implementation Roadmap

A structured roadmap is necessary to implement chrono-omics in the practice of GDM.

Step 1: Biomarker validation. Candidate circadian and omic biomarkers (genomic, proteomic, metabolomic) must be replicated in large, ethnically diverse pregnancy cohorts. For example, emerging markers like placenta-derived circRNAs or metabolic panels show promise, but their expression can vary across populations and platforms [96]. Rigorous validation (analytical and clinical) is therefore essential before clinical use.

Alongside molecular work, simple anthropometric–biochemical tools such as combining the 50 g glucose challenge test with ultrasound measurement of abdominal subcutaneous fat thickness (ASFT > 18.1 mm) also deserve attention. This pragmatic combination predicted GDM with high sensitivity and specificity, and could serve as an early, clinic-feasible triage, to trigger timely chrononutrition counseling and CGM phenotyping [91].

Step 2: Decision-support tools. Once validated, biomarkers and meal timing metrics should be integrated into user-friendly tools (smartphone apps or clinical dashboards) to guide care. Some GDM-specific apps have already been developed, demonstrating improved patient engagement and data-sharing with providers [97]. Future tools could incorporate algorithms that use chrono-omic data (for example, combining CGM profiles with identified biomarkers) to personalize dietary timing and therapy.

Step 3: Interdisciplinary care teams. Effective translation will require training obstetricians, endocrinologists, dietitians and diabetes educators in chrononutrition and omics. Clinicians should learn to interpret omic-based and triage-based risk assessments and advise on meal timing. Multidisciplinary collaboration (e.g., linking geneticists with nutritionists) will be needed so that recommendations on chrono-omics are evidence-based, culturally tailored, and communicated clearly to patients.

#### 6.4.2. Guidelines and Regulatory Considerations

Professional societies and regulators must begin to integrate chrono-omic insights. Diabetes and obstetrics organizations (e.g., ADA, ACOG, Endocrine Society) should review emerging evidence and consider adding recommendations on meal timing to GDM guidelines (for instance, emphasizing earlier dinner or a consistent daily schedule). As an analogy, recent guidelines already use clinical traits (BMI, baseline glucose) for risk stratification [98]. In future, validated omic biomarkers could supplement these measures if shown to predict treatment response or complications. Likewise, pharmacologic guidance might evolve to incorporate circadian principles (e.g., timing of insulin or metformin with respect to chronotype).

Regulatory management will be important for any omics-based test used in pregnancy. In the US, many multi-analyte omic assays would be classified as laboratory-developed tests (LDTs) under CLIA, and FDA is phasing in stricter regulation of LDTs as medical devices [99]. Manufacturers would need to demonstrate analytical validity (accuracy of the assay) and clinical validity (predictive power in pregnant populations). Similarly, in Europe, the new In Vitro Diagnostic Regulation (IVDR) mandates CE marking for complex tests [100]. Regulatory bodies will require evidence of safety and utility—for example, showing that an omics panel used in pregnancy actually improves outcomes without undue risk. Close collaboration between researchers, clinicians, and regulators will be needed to define performance standards for chrono-omic diagnostics in gestation.

#### 6.4.3. Patient Engagement and Education

Communicating chrononutrition to patients requires clear, culturally sensitive education. Complex genetic or metabolic results should be explained in plain language, emphasizing actionable steps (e.g., “eat earlier, avoid late-night snacks”) rather than technical details. Visual tools (meal timing charts, app-based alerts) and multi-language resources can improve understanding and adherence [97,101,102,103]. Importantly, dietary recommendations must be adapted to cultural food practices. For example, an “early dinner” strategy should consider local meal traditions and possible conflicts with family schedules. Qualitative research shows that women from diverse backgrounds often find standard GDM advice insufficient or culturally misaligned [104]. Thus, shared decision-making is key: clinicians should elicit each patient’s beliefs, preferences and daily routine, and co-create a diet plan that respects these factors. Community-based support (peer educators from the same cultural group, group classes) can reinforce chrononutrition principles in a relatable context. By engaging patients and families in goal-setting and education, chrono-omic recommendations can be effectively communicated and implemented, ultimately improving maternal–fetal health.

## 7. Challenges, Gaps, and Future Directions

Precision chrononutrition has great potential to improve GDM care, but ensuring equity is critical. Low-income and rural women often lack reliable internet access or devices, and even when devices are available they may have limited data plans, reducing connectivity [105]. Language, literacy, cultural food practices and work schedules (e.g., shift work) can hinder the adoption of time-based dietary interventions. Historical mistrust in healthcare also disproportionately affects ethnoracial minorities [105]. For example, Hispanic women have some of the highest GDM rates [106] and may face additional cultural and economic barriers to changing meal timing. Precision nutrition approaches based on genetic or metabolic data have mostly been developed in European-ancestry populations [107], risking further disparities unless adapted for diverse groups. Inclusive design (incorporating community input on preferences, beliefs and constraints) is needed so that chrononutrition recommendations are practical across socioeconomic, cultural and occupational contexts [108].

Technological integration poses major challenges. Patient data are often fragmented across paper charts, apps, personal devices and social media resources [106], making it hard to synthesize dietary, circadian and clinical omics information into one view of GDM risk or control. Lack of interoperability standards for EHRs, sensors and laboratory data thwarts aggregation of multi-omics (genomics, metabolomics, microbiome, etc.) with lifestyle and circadian inputs. Advanced analytic tools and AI hold promise to integrate these big data, but “black box” algorithms raise concerns about bias and trust. Studies have found that most AI diabetes models report predominantly white participants and rarely report race/ethnicity [108], so models may not generalize to minority patients. Ensuring explainable AI is vital: clinicians and patients must understand and trust algorithmic advice. Privacy is another concern—detailed omics and continuous monitoring data require robust security and clear consent frameworks. Building trust requires transparency and community engagement [105], as well as algorithmic fairness evaluations to prevent the reinforcement of disparities [108].

Important knowledge gaps remain. There is surprisingly little high-quality data on how meal timing affects maternal–fetal outcomes in diverse GDM populations, and on the molecular circadian rhythms of pregnancy. For example, day–night patterns in placental metabolites or gut microbiota under GDM conditions are poorly understood. The causal mechanisms linking chronodisruption (e.g., shift work or evening chronotype) to adverse outcomes in GDM have not been fully elucidated. We also lack validated multi-omic biomarkers that dynamically reflect clock-related dietary effects. Early efforts such as observational and small interventional trials of time-restricted eating in pregnancy provide hints, but larger cohort studies and randomized trials are needed. In parallel, methods for fusing high-dimensional data must mature—e.g., federated learning or data harmonization platforms—to integrate physiology (circadian metrics), nutrition (diet logs), genomics and clinical records. Addressing these gaps will require longitudinal cohorts of pregnant women (with diverse backgrounds) monitored for circadian behaviors, feeding patterns and metabolic profiles. In the meantime, key practical questions can guide research (see Box 3).

Public health and policy implications should not be overlooked. Current GDM guidelines (e.g., ADA prenatal nutrition therapy) focus on nutrient composition and portion sizes, but rarely address timing. Policy makers and professional bodies (obstetrics, diabetes and nutrition societies) could explicitly incorporate chrononutrition into recommendations, for instance, advising regular breakfast consumption, limiting late-night eating, and aligning snack/meal timing with circadian glucose tolerance patterns. Prenatal programs and dietitian counseling should be updated accordingly. Culturally tailored educational materials and community programs are needed to make chrononutrition advice feasible in real-life settings (e.g., for women in demanding occupations or with variable family meal schedules). Although formal cost–benefit analyses for chrononutrition are not yet available, preventing even a modest fraction of GDM complications through optimized meal timing could offset implementation costs. Moreover, enhancing maternal health now has long-term economic benefits by reducing future type 2 diabetes risk in mothers and offspring.

Looking ahead, the next decade could transform GDM care. We envision AI-enabled decision support embedded in prenatal care: for example, smartphone apps that use wearable sensor data (sleep/wake, activity, continuous glucose monitoring) and personal omics profiles to generate individualized meal timing recommendations. Such a digital coach would learn each patient’s circadian patterns and advise on optimal times for breakfast, lunch, dinner and snacks to minimize glucose excursions. If carefully designed with patient input, AI could help mitigate (rather than exacerbate) health disparities [108]. Federated learning across clinics could continuously improve predictive models while preserving privacy. In practice, prenatal visits might routinely include review of digital food diaries and circadian logs, integrating them with genetic or metabolomic risk markers. Wearable technology will likely become more affordable and widespread, making data-driven chrononutrition feasible even in underserved areas. By combining omics and chronobiology with community-focused implementation, precision GDM care may finally achieve both personalization and equity. To guide future work, we outline key unanswered questions related to chrono-personalized care (see Box 5).

Box 5 **Outstanding questions and future research directions.**

- How can chrononutrition interventions be adapted for women with varying work schedules, cultural diets and socioeconomic constraints?
- What are the key circadian and multi-omic biomarkers of GDM risk and progression, and how can they be validated in large, diverse cohorts?
- Which digital health architectures can integrate EHRs, wearable data and laboratory omics in a privacy-preserving, interoperable way for maternal care?
- What methods (e.g., explainable AI, participatory design) will build trust and ensure bias mitigation in GDM prediction and feedback tools?
- What is the cost-effectiveness of implementing chrononutrition-based strategies in prenatal care programs across different healthcare systems?

## 8. Conclusions

Chrononutrition offers a practical and evidence-based approach to GDM management. Time-based strategies, such as early carbohydrate intake, extended overnight fasting, and structured meal sequencing, can improve glycemic outcomes with minimal burden. When integrated with molecular profiling and real-time monitoring, these approaches move beyond generalized advice toward truly individualized care. Realizing this potential will require solid clinical validation, thoughtful technological integration, and a commitment to equity and cultural relevance. With coordinated efforts across disciplines and systems, precision chrononutrition can shift GDM care toward more effective, personalized, and inclusive solutions, improving outcomes for both mothers and their children.

## Figures and Tables

**Table 2:** Chrononutrition in practice—examples.

Behavioral Target	Practical Strategy	Supporting Evidence
Overnight fasting duration	Aim for an overnight fasting window of ~8–10 h. In individuals with elevated fasting glucose, consider longer fasts (≥12 h) under supervision. Avoid late-night carbohydrate snacks.	Longer overnight fasting (~10–12 h) linked to lower fasting glucose in observational GDM cohorts [80]; however, other studies [81] found extended fasts may worsen glycemic stability, especially when total carbohydrate intake is low or inconsistent. This suggests that overnight fasting duration may need to be tailored to individual glycemic profiles.
Front-load carbohydrate intake	Allocate ~50% of daily carbohydrates to breakfast and lunch; reduce carbohydrate intake at dinner.	Morning carb loading improved glycemic control in one RCT supported by observational and pilot studies [74,80,82].
Minimize evening/night eating	Finish the last meal by early evening and avoid high-carb snacks late at night to extend the overnight fast.	Concentrating calorie intake at night worsens glycemic control. Eating >50% of calories after 7pm → higher fasting and mean glucose [83]; systematic review: later meals and shorter overnight fasting → poorer glycemic outcomes in pregnancy [84].
Choose low-GI, balanced meals	Select low-glycemic-index carbs (whole grains, legumes, etc.) and pair them with protein and healthy fats to slow glucose absorption.	High-glycemic-index meals at lunch/dinner → elevated postprandial glucose [81]; low-GI diet in GDM halved insulin requirement [82].
Sequence food intake	Eat high-fiber vegetables first, then protein, and carbohydrates last.	Sequencing meals this way delays carbohydrate absorption, moderates glucose spikes, and improves time-in-range [40,77].

## Data Availability

All data generated or analysed during this study are included in this published article and its Appendix A.

## Appendix A

**Table A1:** Chrononutrition strategies across pregnancy trimesters in the context of GDM.

Trimester	Applicability to GDM	Key Physiologic Point	Food Group and Timing Recommendation	Evidence Notes
First trimester (0–13 wk)	Not applicable (GDM rarely diagnosed at this stage)	Insulin resistance low Nausea, variable appetite	Focus on nutrient sufficiency: vegetables (non-starchy), lean protein (lean meat, eggs, tofu, legumes, dairy), carbohydrates (whole grains, low-GI fruits), nuts, seeds, healthy fats. Avoid prolonged fasting. If nausea, small frequent meals. Do not start the day with a carbohydrate-only meal; prefer vegetables/protein first.	No evidence; GDM not identified this early
Second trimester (14–27 wk)	Primary window for actionable timing strategies	Rising insulin resistance from placental hormones; appetite improves	Front-load carbohydrates earlier in the day but do not start the day with carbs alone. Practical rule: breakfast = protein + vegetables (then carbs if needed). Emphasize whole grains, legumes, non-starchy vegetables, lean protein, unsweetened dairy. Nuts, seeds, and healthy fats useful as carbs “buffers” in meals. Avoid SSBs and large late-night carb loads.	No trimester-specific studies. Evidence from RCTs, pilot trials, and cohorts and systematic review in women with GDM [40,52,70,74,77,80,82,83,84]
Third trimester (28 wk–delivery)	Ongoing GDM management	Peak insulin resistance Gastric emptying slows Higher risk of postprandial hyperglycemia Fetal growth acceleration	Continue 2nd-trimester strategies: Front-load carbs earlier, vegetable → protein → carb sequencing, limit late-evening carbs. Avoid late-night snacking unless medically required (e.g., insulin). Continue nuts, seeds, and healthy fats as meal addition—they can buffer carb absorption. Individualize overnight fasting length and monitor with CGM when changing patterns. Avoid prolonged caloric restriction.	No trimester-specific studies. Evidence from RCTs, pilot trials, cohorts and systematic review in women with GDM [40,52,70,74,77,80,82,83,84]

Abbreviations: SSB—sugar-sweetened beverage; RCT—randomized controlled trial.
